# The Effects of a Novel Treatment for Hemianopic Dyslexia on Reading, Symptom Load, and Return to Work

**DOI:** 10.3390/brainsci14030259

**Published:** 2024-03-06

**Authors:** Georg Kerkhoff, Antje Kraft

**Affiliations:** 1 Department of Clinical Neuropsychology & Neuropsychological Outpatient Unit, Saarland University, P.O. Box 15 11 50, D-66041 Saarbrücken, Germany; 2 Zentrum für Ambulante Neuropsychologie und Verhaltenstherapie (ZANV), Schleiermacherstraße 24, D-10961 Berlin, Germany; kraft@zanv.de

**Keywords:** brain damage, visual field defect, dyslexia, treatment, return to work

## Abstract

Reading disorders are frequent in homonymous hemianopia and are termed hemianopic dyslexia (HD). The existing treatment methods have shown improvements in reading speed, accuracy, and eye movements during reading. Yet, little is known about the transfer effects of such treatments on functional, reading-related tasks of daily life, e.g., reading phone numbers, finding typing errors or text memory. In addition, little is known about the effects on symptom load and return to work. Here, we examined a new reading therapy entailing three different methods—floating text, rapid serial visual presentation (RSVP) of single words, and the moving window technique—and evaluated their efficacy. Twenty-seven chronic HD patients were treated in a baseline design with treatment-free intervals before and after a treatment period of several months. HD was assessed with a battery of reading tests and a questionnaire about subjective symptom load at four time-points. Patients received all three reading therapies over several weeks. The results show significant and stable improvements during treatment within all measures. Approximately 63% of treated patients returned to work after the therapy. We concluded that our novel HD treatment led to widespread and lasting improvements in reading performance, generalized to functional reading tasks and reduced symptom load, and the majority of patients were able to return to work.

## 1. Introduction

Homonymous visual field defects (VFDs) are one of the most frequent consequences after brain damage and occur in about 20–50% of patients with cerebrovascular disorders [1,2,3,4,5,6]; they can occur also after traumatic brain injury [7]. Spontaneous field recovery is present in the first 2–3 months post-lesion in up to 40% of the patients with a stable etiology such as stroke [8]. However, after six months, spontaneous recovery of the visual field is extremely unlikely [8]. Patients with homonymous VFDs suffer from three main impairments: a visual search deficit in the blind and intact visual field [9], a reading disorder [10], and a contralesional shift of the perceptual midline (line bisection error [11]). The impact of VFDs and their related visual impairments on daily life is well documented (see [1] for an overview), yet there is insufficient evidence for the development of effective therapies for these disorders [10]. Return to work has been found to be especially difficult in patients with left-sided VFDs [12].

Acquired reading disorders are the most frequently reported problem in patients with VFDs (>40%, [6,13]). Moreover, reading disorders are significantly associated with mental fatigue, anxiety, and depression [6] and thus impair daily functioning. While vision-related reading disorders can have multiple causes, visual field defects are probably the most common. Visual field sparing (<5° on the blind side) occurs in 70% of all stroke patients with VFDs [2]. This is critical for reading, as the central visual field (+/−5° around the fovea) is crucial for fluent reading. Visual acuity and form recognition within this area are sufficient for letter recognition (“perceptual reading window” [14]). Consequently, slow, error-prone reading is typical for VFD patients with less than 5° field sparing on the blind side [2]. This phenomenon is termed hemianopic dyslexia (HD) [15]. In contrast to HD, pure alexia is characterized by extremely slow reading (<25 words per minute) and often also by letter-by-letter (LBL) reading [15]. It is important to note that HD has to be differentiated from neglect dyslexia—another reading disorder. Neglect dyslexia is characterized by numerous omissions and substitutions of syllables and words on the contralesional side of a text or a single word [16,17,18] and correlates with contralesional omissions in cancellation tasks [17]. The latter can be used to differentiate HD from neglect dyslexia (see below) and a specific trunk rotation maneuver can disentangle real field defects from neglect-induced, artificial field defects [19]. 

Therapy for HD most often entails the oculomotor compensation of the reading deficit that arises from the loss of parafoveal visual field regions that have sufficiently good visual acuity to identify letters and syllables. The most effective strategies for reading training for HD use an “optokinetic” approach [10,20,21]. Here, letters, syllables, words, and numbers are presented in a single text line which floats from the right to the left side on a computer screen, while the patient is instructed to read the words in the middle of the screen (floating text). The moving words induce pursuit eye movements on the side of motion and an optokinetic nystagmus on the opposite side [20]. Another effective technique is the training of saccadic eye movements using static words [22]. Several treatment studies have shown that these two types of treatment significantly improve reading speed, reduce reading errors, and diminish the number of eye fixations during reading (for review see [1,10,23]). However, reading techniques are culture dependent [24,25,26]. In cultures that read from left to right, the motion should be from right to left; in those that read from right to left, the motion should be left to right; and in those that read from top to bottom, the motion should move upwards. Moreover, for patients with right-sided VFD, it was shown that this specific patient group benefited from a vertical training procedure in reading tasks, while horizontal training improved reading speed in patients with left VFD [27]. 

While the floating and saccadic reading therapy for HD have been validated in several studies and represent a viable treatment for HD, little is known about its transfer effects to daily life [1]). In a broader sense, we know almost nothing about the performance of patients with HD in reading-related functional tasks, their subjectively perceived symptoms related to reading in daily life and whether they are able to return to work after treatment. Many patients with HD complain about increased fatigue during reading [6], and that reading is so laborious and effortful for them that after reading some lines, they have almost forgotten what they read. It is interesting to note that the most frequent etiology of HD is a left posterior cerebral artery infarction [21,28,29], which in turn, often causes verbal memory impairments [30] due to the anatomical overlap of the regions devoted to reading, verbal learning and memory. Hence, verbal memory during reading seems to be a critical problem in HD but has not been assessed in these patients. Moreover, many patients with HD indicate that “searching or scrolling” a text quickly when looking for a certain passage, number or typing error is often difficult or even impossible for them after their brain damage. This indicates that the rapid searching of a text is also a severe problem in HD. Beyond this, difficulties in comparing and typing long numbers like phone numbers or bank account numbers are other frequent complains in individuals with HD. Hence, there seem to be multiple daily life impairments related to reading difficulties in individuals with HD. In addition, we also face many different reading requirements in our increasingly “digitized” or even “virtual” world, e.g., reading single words, numbers or phone numbers, and whole texts; searching throughout a text and/or checking for errors or certain information in a text; and memorizing written information for later recall, to name only a few. Furthermore, we read not only on printed paper, but increasingly more often on a computer screen, a tablet, a smart phone, messenger or other electronic visual displays (e.g., fitness watches, displays on machines, electronic traffic signs, and timetables, etc.). These different aspects of reading and functional reading tasks are important for our daily lives and also relevant for the vocational reintegration of individuals with HD because their jobs often require many of these capacities. However, little is known about these functional aspects of reading in HD, and to our knowledge, there is no research on their treatment. 

In the present study, we therefore evaluated the effects of a novel combination of three different reading techniques (floating text, rapid serial presentation, and moving window technique) in a sample of individuals with mostly chronic HD (>four months). We used a battery of basic clinical and functional reading tasks—partially paper-based and on a computer screen—to evaluate reading performance and quality of life (subjective symptom load related to reading performance) in various daily life settings. This was also performed because two subsequent Cochrane analyses analyzing the effects of neuro-visual rehabilitation in patients with hemianopia and reading disorders concluded that there is insufficient evidence for any positive treatment effect on functional aspects of vision in daily life, including reading [31,32]. Moreover, it is unknown whether and how patients with HD can return to their prior work after rehabilitation. We therefore sought to evaluate the following research questions in our study:

(1) How do individuals with HD perform in basic clinical reading tasks? How are paper-based reading and reading on a computer screen related to each other?
(2) Does the novel reading treatment lead to stable and significant improvements within the reading test battery? Does it transfer to functional reading tasks and reduce symptom load related to reading?
(3) How is vocational reintegration in individuals with HD?

## 2. Materials and Methods

### 2.1. Patient Recruitment

The sample of 27 patients (see Table 1) were being treated as outpatients in the university neuro-psychological outpatient unit at the Saar University in Saarbrücken/Germany (under the care of GK). Here, they received long-term neuro-psychological treatment, which was covered by private or public health insurance. The patients underwent a routine assessment of neuro-visual and cognitive tests, which are described in the Section 2.2. They were subsequently enrolled in the study when they fulfilled the following criteria:

- Significant reading impairment in the first 2 reading tests of the test battery (see Table 2, tests 1 and 2).
- No evidence of pure alexia based on 3 tests for differential diagnosis (vertical reading task, letter-by-letter-reading, reading speed > 25 Words per Minute, see Section 2.2.2).
- No evidence of visual neglect (based on conventional neglect screening tests [33]. If it was unclear whether a field defect was caused by neglect we used the trunk-rotation method suggested by Nyffeler et al. [19] to disentangle real from pseudo visual field defects (the latter influenced by neglect).
- No premorbid (i.e., developmental) dyslexia according to medical records, nor dementia according to medical records, and no aphasia (based on the Aachen Aphasia Test AAT, [34]).
- Availability for regular treatments in the outpatient unit over a period of at least several weeks.

No ethical approval was necessary since all patients sought and received a regular, standard treatment of their reading disorder in the outpatient unit.

### 2.2. Assessments

#### 2.2.1. Neuro-Visual Screening Tests

Visual acuity was measured with high-contrast letter acuity charts for the near (0.4 m distance, Oculus Nahleseprobe, Wetzlar, Germany) and far (6 m distance, Oculus acuity letter chart, Fronhäuser, München, Germany) viewing conditions. A number cancellation task and horizontal line bisection were used to test for visual neglect (described in [33]). It requires the cancellation of 20 single digits embedded in 180 single digits serving as distractors which are displayed on A4-sized white paper. Horizontal line bisection was performed manually by the patient using a pencil. Cut-off scores are available for both tests from 40 healthy control subjects [33]. Visual campimetry was performed to test the central 70° (horizontal extension) × 50° (vertical extension) of the visual field using the Eyemove software package Version 3 [35], URL, https://www.medicalcomputing.de, assessed on 26 February 2024. In some cases, additional perimetry with the centerfield perimeter (Oculus, Wetzlar, Germany) was performed monocularly, especially to detect small paracentral or diffuse scotomas. Neuro-visual screening tests were performed at every assessment time point, but the results typically did not change over time. Therefore, they are only reported for the first baseline assessment in Table 1. 

#### 2.2.2. Differential Diagnosis of HD from Pure Alexia

To differentiate HD from pure alexia, 3 tasks were used: (a) vertical and horizontal reading of 8-letter words. HD patients are typically impaired in horizontal reading (due to the horizontal parafoveal field loss), while pure alexics are impaired in both reading directions due to slow letter processing in every reading direction. If reading was impaired in both reading directions, patients were excluded from the sample. (b) Letter-by-Letter (LBL) reading. LBL reading is a clear sign of pure alexia [15], thus, if LBL reading was present, the patient was excluded from the sample. (c) Pure alexics show a reading speed of <25 words per minute (WpM), while HD patients typically have a reading speed of >25 WpM [15]. Thus, if the reading speed was below 25 WpM, the patient was excluded from the sample.

#### 2.2.3. Reading Test Battery and Subjective Symptom Load Questionnaire

A battery of 6 reading tests (see Table 2) was used to measure the initial impairment and improvement during therapy. Most tests had multiple parallel versions to avoid test repetition effects due to memorizing previously read test material. In addition, a questionnaire was read to the patients (see Appendix A) to evaluate the subjective impairments related to HD, other neuro-visual problems, and their potential change throughout the treatment. The questions were read orally to the patient who responded to every question using a 5-point scale on whether the statement held true for them. Importantly, the subjects did not read the questionnaire by themselves to avoid a confounding of results by visual or reading problems. The rating in the questionnaire included the following gradations: 0 = not at all, 1 = rarely, 2 = partly, 3 = mostly, 4 = totally. Example: “I have had difficulties in reading the newspaper”. When the patient indicates “mostly”, the score for this question would be 3. This procedure was performed for all items, and the summed score was used for the analyses. 

##### Detailed Description of Reading Tests

Test 1 from Table 2 is a simple paper-based reading test that exists in 6 parallel forms and was used in a previous study with HD patients [13], and in patients with left visual neglect [18,24]. The 6 parallel reading tests (test 1 in Table 2) were standardized using a sample of 60 healthy subjects between the ages of 17 and 68 (AM = 39.9; SD = 15.57) from three age groups (17–30 years, 31–50 years, and 51–68 years). This was conducted to determine the standard values for the number of errors and for reading speed. Each of these age groups consisted of ten women and ten men. All subjects had a good or corrected near visual acuity of 0.9. 

Tests 2–6 from Table 2 are part of a standardized battery of reading tests developed to assess different aspects of reading performance in individuals with visually related reading disorders, such as HD, neglect dyslexia, and pure alexia, or reading disorders resulting from oculomotor deficits, reduced contrast sensitivity, Balint–Holmes syndrome or other neuro-logical conditions. The battery comprises 18 different subtests (described in detail elsewhere [25]): 15 subtests evaluate different aspects of single-word or text reading; 3 subtests evaluate the reading of numbers or a visual search of numbers. While 13 tests are designed to test a certain aspect of reading (e.g., reading compound words or text under different contrast conditions), the other 5 subtests are designed to assess more functional aspects of reading (such as subtests 4–6 in the present study). For each subtest, 3 parallel versions of identical length (100 words in the reading tests, comparable number of digits in the numerical tasks) and comparable task difficulty were developed and standardized in a sample of 40 healthy subjects (age 20–75 years; 20 males, 20 females). 

##### Retest Reliability

For all tests of the reading battery including the subjective symptom load questionnaire, we computed the retest reliability coefficients from the measurements at Base 1 and Base 2 in the present clinical sample. The time interval between the two measurements was 65 days (mean). The coefficients (see Table 2) showed excellent retest reliability scores (both for Pearson and Spearman coefficients); the repeated measurements with parallel versions of these reading tests and the subjective symptom load questionnaire also showed excellent reliability scores. Moreover, these tests have been extensively used in our research unit in numerous previous studies and were found to be sensitive and reliable measures in clinical practice for patients with HD [13,22,23] or other peripheral visual reading disorders (i.e., neglect dyslexia). Hence, our reading test battery includes classical measures of text reading where typically “words per minute read correctly” or time and errors are counted [10,22] and also tests additional aspects of reading under other conditions (e.g., single-word reading). Moreover, it also includes functional reading tasks (such as text memory, finding typing errors in a text, reading and typing phone numbers), which, to date, have not been used to assess patients with HD. Furthermore, this test battery was used in a clinical study [37] and has proven to be able to capture different aspects of reading impairment in patients with HD and other reading disorders. It has also been proven to be able to monitor treatment progress after reading therapy which is the focus of the present study.

### 2.3. Treatments

**Figure 1:**
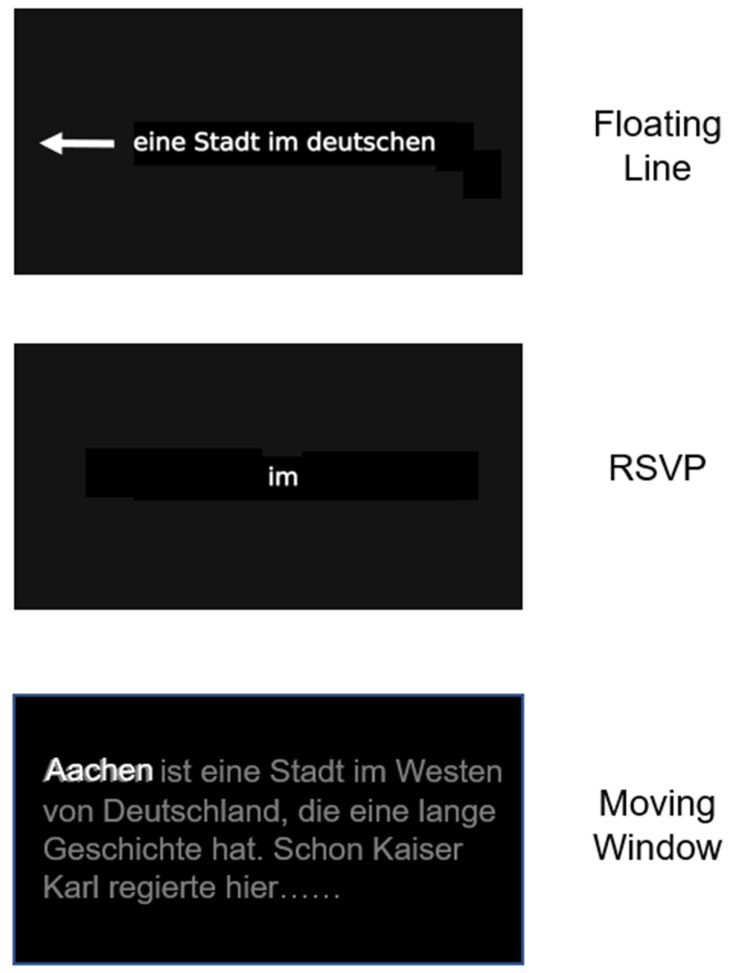
Schematic illustration of the 3 reading therapy techniques used in the study. During floating line therapy, only a single line of text moves from right to left through the middle of the screen (as indicated by the white arrow, which was not visible during therapy). During RSVP, single words are presented in the center of the screen. During the moving window therapy, several lines of a complete text were presented, but only one word was printed in white which was the word that should be read out aloud. The next word was activated by a button-press from the patient; the next word then appeared (also in bright white), while the previously read word turned dark grey. All other words of the text were barely visible, because they were shown in dark grey to give some spatial orientation in the line and on the screen, but at the same time reduce visual crowding arising from other words. See text for more details.

The three types of treatment used in this study are part of the Neuro-Vision Training (NVT), version 1 software package (URL: https://www.neuro-vision-training.com, accessed on 26 February 2024). The idea was to implement those reading techniques into the software that have either been shown to be effective for patients with visual reading disorders resulting from acquired brain damage (the floating text line, see below [23]), or have been shown to be useful in training reading in healthy subjects (the RSVP technique [38] and the moving window technique [39]). The treatments were given to every patient for the same amount of time in every therapy session. Hence, all three reading techniques were trained to the same extent. This was performed with the aim of maximizing the benefits of the reading test battery and the subjective symptom load list through their differential requirements during reading. Below, these 3 techniques are described in more detail, and Figure 1 schematically shows the visual display of these reading techniques as they appear on the computer screen.

Floating text line—Here, a single line of text floats from right to left through the middle of the computer screen with an adaptable speed. The patient was instructed to read the words aloud in the middle of the text line and then move the eyes again to the right to read the word(s) that appear next. Therapy started with short, high-frequency words (3–5 letters), followed by increasingly longer words and short sentences (3–4 words long). Finally, complete texts were read using this technique. This is the reading technique that most HD treatment studies have used so far (cf. [23]).

Rapid serial visual presentation (RSVP) of single words—Here, single words were presented sequentially in a central window on the computer screen. The patient was instructed to read them aloud as correctly and quickly as possible and to initiate presentation of the next word by pressing the space bar. Hence, the patient controlled the speed of the presentation of the single words by pressing a button. RSVP is a well-known technique that has been studied intensively in healthy subjects [38]. It promotes faster reading, mostly because few if any saccadic eye movements have to be made in contrast to text reading. In addition, it reduces “crowding” of multiple visual stimuli because only one word is presented. Visual “crowding” can significantly impair reading in individuals with brain damage [40]. We therefore hypothesized that RSVP could also be beneficial for our patients because it reduces crowding during reading. Moreover, to the best of our knowledge, RSVP has not been used in the treatment of HD.

Moving window technique (MWT)—Here, words and several lines of text appear in the correct position on the computer screen, but only one word is visible at a time, because the other words are visually suppressed and barely visible. This technique has been extensively used in reading research with healthy subjects (for review see [39]). It promotes line reading but avoids the simultaneous presence of multiple words on the screen. Hence, it also reduces “crowding” phenomena that arise when reading a conventional text with many words where all the words are visible simultaneously. In contrast to the other two reading techniques, the moving window technique corresponds most closely to the “normal” reading of a text with multiple lines. To our knowledge, this technique has also not been used in the treatment of HD.

General aspects of the reading treatment—All three methods were used in equal proportions within each therapy session. Each session started with the floating text (20 min), followed by reading with the RSVP technique (20 min), which was followed by reading with the moving window technique (20 min). A great variety and number of different reading texts (>80) were available for therapy in all three reading techniques in the Neuro-Vision Training software package. Every patient started their therapy on an easy reading level in the 3 different techniques, consisting of short, high-frequency and familiar words (from word counts). The patient sat comfortably and centered at 0.5 m from a 20″ computer screen. They were encouraged to read aloud the words or numbers appearing in the middle of the screen as correctly and as quickly as possible. Errors were immediately corrected by the therapist, and the erroneous word was read again. When the reading speed improved, the speed of presentation was gradually increased in the software by the therapist (in the MWT and floating line technique). During RSVP, the patient was encouraged to read the single words appearing in the center of the screen as quickly as possible without omitting syllables on one side (i.e., the blind side). After reading one word, they should quickly press the space bar to trigger the appearance of the next word. It takes some time to adapt patients to this self-paced modus of reading, but all patients learned it quickly within the first few sessions and then could increase their reading speed. Head movements during reading in front of the PC screen were discouraged in all three reading techniques because they do not improve the eye movements necessary for better reading. Instead, patients were told that they should read using eye movements without moving their head. All patients wore their necessary glasses for the distance of 0.5 m to have optimal visual acuity.

**Figure 2:**
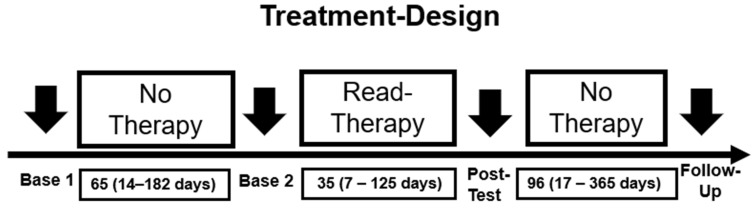
Design of the study. Bold arrows indicate points of assessments. The time intervals between these time points are indicated below in the boxes in days (mean, range).

Other treatments—No other treatments (e.g., visual, reading or cognitive) were performed during the reading therapy phase (see Figure 2). Likewise, no other treatments were given to the patient before or after the reading therapy (i.e., in the baseline period and the follow-up period). However, all patients continued consulting their medical doctors (general practitioner and/or neurologist) on a regular basis (usually once per quarter) to discuss the medical aspects of their disease including their (potential) medication. Medication was held constant by the medical doctors throughout the whole treatment design for their patients. No therapy for the reading disorder nor any other behavioral treatment was given by the medical doctors to the patients.

### 2.4. Design and Statistics

#### Patient Recruitment and Enrollment

Patients were recruited through our neuro-psychological outpatient unit at Saarland University. Here, patients with any neuro-psychological impairment due to acquired brain damage which dates back no more than 5 years can receive specific neuro-psychological assessment and therapy. The patients in this study all contacted the office of our outpatient unit via phone, email or mail with the aim of obtaining therapy for their reading disorder. All patients provided their neurological, neuro-radiological and neuro-psychological findings (as far as available) in advance. These were read by GK in order to decide whether there was an indication for neuro-psychological therapy. An initial examination appointment was then arranged, at which a standardized anamnesis of the neuro-visual [13] and cognitive complaints took place. The neuro-visual tests and neuro-psychological tests for differential diagnosis (see Section 2.2.1 and Section 2.2.2) were then carried out to determine which disorders were present. If impairments were present in the first two tests of the reading test battery, the complete test battery including the subjective symptom burden list was carried out. At the end of these tests, reading therapy was offered to every patient who fulfilled the inclusion criteria of this study. The patients with reading disorders that were excluded by the exclusion criteria were also offered reading therapy but were not included in this study. An application for reimbursement was then immediately submitted to the relevant health insurance company or other payer (employers’ liability insurance association) on behalf of the patient. At the end of this first appointment, it was agreed that the reading therapy would begin as soon as the declaration of cost coverage was received. At the second assessment appointment, all reading assessments and the questionnaire were repeated. Following this, therapy began immediately as described above. Importantly, no bio-ethical approval was necessary for this procedure as it is genuine clinical practice and represents the standard procedure for the treatment of neurological patients suffering from neuro-psychological disorders in Germany. However, the practitioner(s) must be licensed and have all the necessary qualifications as a psychological psychotherapist and clinical neuropsychologist with further chamber training. This is the case for both authors (GK, AK). Both are certified members of their respective chambers of psychotherapists (Saarland, Berlin, Germany) with the necessary additional training in clinical neuropsychology. As all patients in this study had a corresponding indication for neuro-psychological treatment, all patients were offered appropriate treatment, which was accepted by all. The planning of the appointments, their frequency and their exact timing was then carried out individually with each patient. All patients agreed to the treatment offered. They were informed that they could interrupt or discontinue treatment at any time. In summary, it can be said that this procedure represents the standard procedure for outpatient clinical neuro-psychological diagnostics and therapy for patients with acquired brain injury in Germany. No separate ethics approval is required for this.

All patients were assessed and treated in a single-subject baseline design with 4 assessment time points (Base 1, Base 2, Post-Test, Follow-Up; see Figure 2). The first baseline test (Base 1) was used to enroll the patients, perform all assessments, and introduce the reading therapy in detail. Before starting reading therapy, however, an application (to the health insurance) must be made (by GK) for the costs of treatment to be covered. This usually takes 3–6 weeks. This period was used as the first (pre-treatment) baseline phase. As soon as the cost coverage was confirmed, the patients were called in for the second baseline measurement. Here, all assessments (including the neuro-visual screening tests) were carried out again with the respective parallel versions of the tests (if available). Reading therapy began immediately after the second baseline assessments. The amount of reading therapy given to each patient varied, although we tried to give as much treatment as possible in every case. It critically depended on 2 factors: (a) the amount (hours of therapy) that was covered by the health insurance, and (b) the time (weeks) every patient could attend our outpatient unit for therapy. Since 16 out of the 27 (59%) patients came from regions in Germany that were too far away for daily commuting to the outpatient unit in Saarbrücken, these patients came for a limited number of weeks and received their whole reading treatment in these weeks. The remaining 11 patients (41%) came from the nearby Saarland region and their reading therapy was spread over a longer time period. This is the reason for the variability in the time intervals of the different treatment phases (see Figure 2). No patient volunteered to interrupt or cancel treatment. Likewise, in no case was the reading therapy discontinued by the therapist, i.e., because of missing improvements. Every therapy session lasted 60 min. All patients were familiar with a computer at home.

After the end of the treatment, a post-test with all assessments (including the neuro-vision screening tests) was performed again and then the patients were discharged home. Several months later, a follow-up test was arranged with identical assessments.

Since the sample was small and permitted no meaningful conclusions on the normality of the data distribution, non-parametric testing was applied throughout. The data were analyzed with nonparametric Friedman one-factorial analysis of variance over the 4 time points. Subsequent post-tests were performed using Wilcoxon tests between time points. The level of significance (alpha) was 0.05 (two-tailed), which was adapted for the number of performed comparisons (Bonferroni-corrected). Hence, alpha was divided by 3 when 3 paired comparisons were performed. In addition, Pearson, Spearman rank, and point-biserial correlation coefficients were computed.

## 3. Results

### 3.1. Basic Clinical Tests

#### 3.1.1. Reading Text on Paper

**Figure 3:**
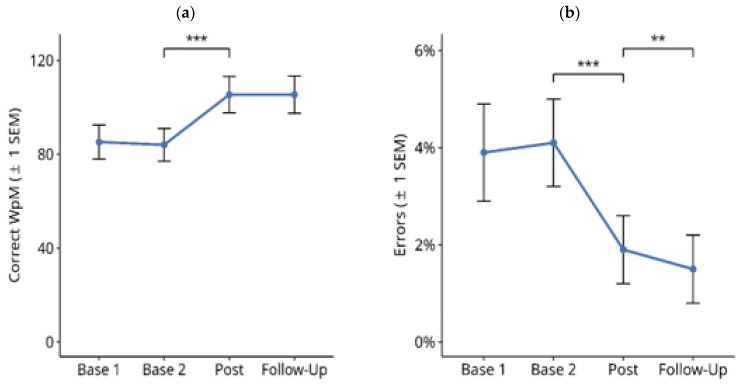
Effects of reading therapy for reading text on paper in 27 patients with hemianopic dyslexia (HD) throughout the course of the treatment study. (**a**) Number of correctly read words per minute (Correct WpM) +/− 1 standard error of the mean (SEM). (**b**) Readings errors (%) in the same task. The brackets and asterisks indicate significant statistical differences between conditions using two-tailed Wilcoxon test; (**) *p* ≤ 0.01, (***) *p* ≤ 0.001.

Figure 3 shows the results for reading text on paper throughout the treatment study. A Friedman test over the four time points showed a significant difference for the number of correctly read words per minute (Figure 3a; X^2^ = 4.685, df = 3, *p* < 0.001). Subsequent paired comparisons using Wilcoxon tests showed no significant difference between the Base 1 and Base 2 values (Z = −1.768, *p* = n.s.), nor between the post-test and follow-up values (Z = −0.212, *p* = n.s.). However, there was a significant improvement in reading speed from Base 2 to post-test (Z = −4.238, *p* < 0.001). The mean improvement from the averaged pre-treatment baselines (Base 1 and Base 2) to the averaged post-treatment measures (post-test and follow-up) in this period was 20.8 correct WpM.

Similar results were obtained for the percentage of reading errors (Figure 3b): there was a significant difference between the four time points (Figure 3b; X^2^ = 58.987, df = 3, *p* < 0.001). Subsequent paired comparisons using Wilcoxon tests showed no significant difference between Base 1 and Base 2 values (Z = −1.787, *p* = n.s.). There was a significant reduction in reading errors from Base 2 to post-test (Z = −4.141, *p* < 0.001). The mean reduction in reading errors from the averaged pre-treatment baselines (Base 1 and Base 2) to the averaged post-treatment measures (post-test and follow-up) in this period was 2.6%.

Furthermore, there was a numerically small, but statistically significant reduction in reading errors from the post-test to the follow-up time points (Z = −3.125, exact *p* < 0.002). Overall, there were only a few reading errors.

#### 3.1.2. Reading Text on PC screen

**Figure 4:**
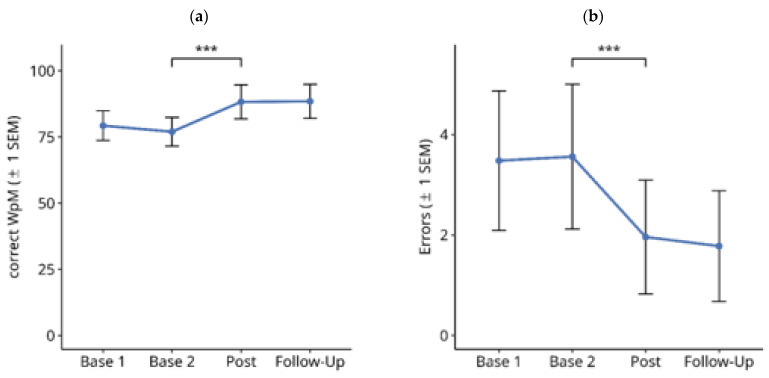
Effects of reading therapy on Text Reading on computer screen in 27 patients with Hemianopic dyslexia (HD) throughout the course of the treatment study. (**a**) Number of correctly read words per minute (Correct WpM) +/− 1 standard error of the mean (SEM). (**b**) Readings errors in the same task. The brackets and asterisks indicate significant statistical differences between conditions using two-tailed Wilcoxon test; (***) *p* ≤ 0.001.

Figure 4 shows the results for text reading on a computer screen throughout the treatment study. Friedman tests over the four time points showed a significant difference in the number of correctly read words per minute (Figure 4a; X^2^ = 33.172, df = 3, *p* < 0.001). Subsequent paired comparisons using Wilcoxon tests showed no significant difference between Base 1 and Base 2 values (Z = −2.328, *p* = n.s.), and between the post-test and follow-up values (Z = −0.587, *p* = n.s.). However, there was a significant improvement in reading speed from Base 2 to the Post-Test (Z = 3.518, *p* < 0.001). The mean improvement in reading speed from the averaged pre-treatment baselines (Base 1 and Base 2) to the averaged post-treatment measures (post-test and follow-up) in this period was 9.8 correct WpM.

Similar results were obtained for the number of reading errors (Figure 4b). There was a significant difference between the four time points (Figure 4b; X^2^ = 40.389, df = 3, *p* < 0.001). Subsequent paired comparisons using Wilcoxon tests showed no significant difference between Base 1 and Base 2 values (Z = −0.229, *p* = n.s.). There was a significant reduction in reading errors from Base 2 to Post-Test (Z = −3.441, *p* < 0.001). The mean reduction in reading errors from the averaged pre-treatment baselines (Base 1 and Base 2) to the averaged post-treatment measures (post-test and follow-up) in this period was 1.15%. Furthermore, there was no significant change in reading errors from the post-test to the follow-up time points (Z = −2.236, exact *p* = 0.025, n.s).

#### 3.1.3. Single-Word Reading on Computer Screen

**Figure 5:**
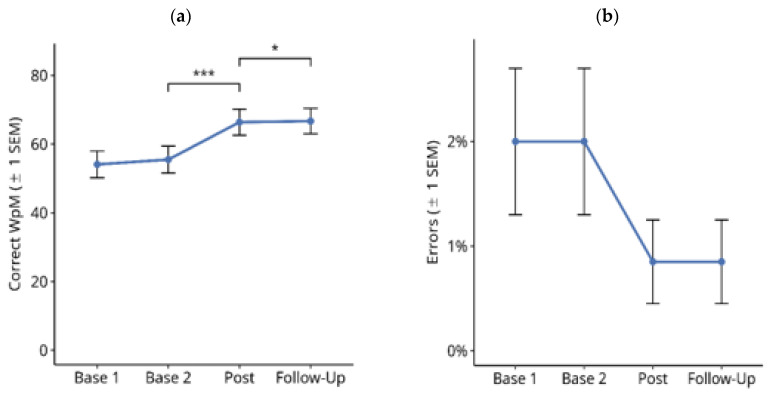
Effects of reading therapy on Single Word Reading on computer screen in 27 patients with Hemianopic dyslexia (HD) throughout the course of the treatment study. (**a**) Number of correctly read words per minute (Correct WpM) +/− 1 standard error of the mean (SEM). (**b**) Readings errors in the same task. The brackets and asterisks indicate significant statistical differences between conditions using two-tailed Wilcoxon test; (*) *p* ≤ 0.05, (***) *p* ≤ 0.001.

Figure 5 shows the results for single-word reading on a computer screen throughout the treatment study. A Friedman test over the four assessment points showed a significant difference in the number of correctly read words per minute (Figure 5a; X^2^ = 42.985, df = 3, *p* < 0.001). Subsequent paired comparisons using Wilcoxon tests showed no significant difference between Base 1 and Base 2 (Z = −1.113, *p* = n.s.). There was a significant improvement in reading speed from Base 2 to post-test (Z = −3.628, *p* < 0.001). The mean improvement from the averaged pre-treatment baselines (Base 1 and Base 2) to the averaged post-treatment measures (post-test and follow-up) in this period was 11.8 correct WpM.

Furthermore, there was a significant though numerically very small increase in reading speed between the post-test and the follow-up time points (Z = −2.490, exact *p* = 0.013).

Similar results were obtained for the number of reading errors (Figure 5b): there were significant differences between the four time points X^2^ = 20.167, df = 3, *p* < 0.001). Subsequent paired comparisons using Wilcoxon tests showed no significant difference between Base 1 and Base 2 values (Z = 0, *p* = n.s.), and between the post-test and follow-up values (Z = 0, *p* = n.s.). There was no significant change in reading errors from Base 2 to post-test (Z = −2.350, exact *p* = 0.019, n.s.). The mean reduction in reading errors from the averaged pre-treatment baselines (Base 1 and Base 2) to the averaged post-treatment measures (post-test and follow-up) in this period was 1.2%.

Reading speeds (correct words per minute) in the reading tests on paper and on a computer screen test correlated significantly with r = 0.814, *p* < 0.001 (Spearman correlation coefficient, two-tailed). Moreover, the reading errors also correlated significantly with each other in both tasks with r = −0.637, *p* < 0.01 (Spearman coefficient).

### 3.2. Functional Reading Tests

#### 3.2.1. Text Memory

**Figure 6:**
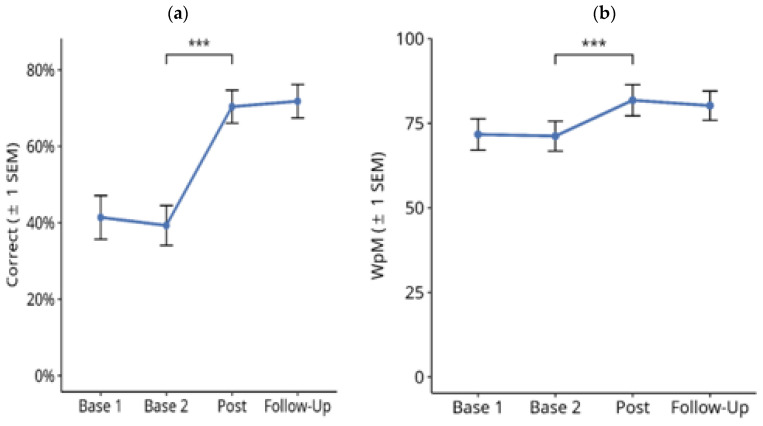
Effects of reading therapy on the number of correctly remembered information in Text Memory test (**a**) and words per minute read in the same task (**b**). The brackets and asterisks indicate significant statistical differences between conditions using two-tailed Wilcoxon test; (***) *p* ≤ 0.001.

Figure 6 shows the results for the percentage of correctly remembered text information in the memory task (a) and the number of words read per minute in the same task (b). Reading errors were not counted because the focus was on text memory performance. A Friedman test over the four time points showed a significant difference in correctly remembered text information (Figure 6a; X^2^ = 49.967, df = 3, *p* < 0.001). Subsequent paired comparisons using Wilcoxon tests showed no significant difference between Base 1 and Base 2 values (Z = 1, *p* = n.s.), and between the post-test and follow-up values (Z = −1.414, *p* = n.s.). However, there was a significant improvement in text memory from Base 2 to post-test (Z = −3.974, *p* < 0.001). The mean improvement from the averaged pre-treatment baselines (Base 1 and Base 2) to the averaged post-treatment measures (post-test and follow-up) in this period was 30.7%.

In addition, an increase was found for the number of words read per minute in the same task: there was a significant difference over the four time points (Figure 6b; X^2^ = 33.847, df = 3, *p* < 0.001). Subsequent paired comparisons using Wilcoxon tests showed no significant difference between Base 1 and Base 2 values (Z = −0.649, *p* = n.s.), and between the post-test and follow-up values (Z = −0.057, *p* = n.s.). However, there was a small but significant increase in reading speed from Base 2 to post-test (Z = −4.085, *p* < 0.001). The mean improvement in reading speed from the averaged pre-treatment baselines (Base 1 and Base 2) to the averaged post-treatment measures (post-test and follow-up) in this period was 9.6 WpM.

#### 3.2.2. Finding Typing Errors

**Figure 7:**
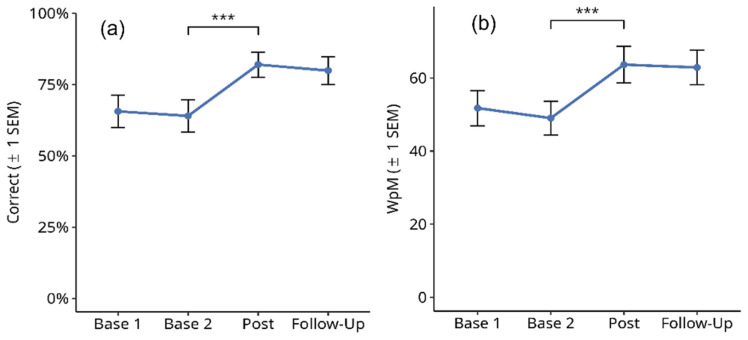
Effects of reading therapy on the number of correctly detected typing errors in a text (**a**) and words per minute read in the same task (**b**). The brackets and asterisks indicate significant statistical differences between conditions using two-tailed Wilcoxon test; (***) *p* ≤ 0.001.

Figure 7 shows the results for the percentage of correctly detected typing errors in a text (a) and the number of words read per minute in the same task (b). Reading errors were not counted because the focus was on the detection of typing errors. A Friedman test over the four time points showed a significant difference in the number of detected typing errors (Figure 7a; X^2^ = 32.773, df = 3, *p* < 0.001). Subsequent paired comparisons using Wilcoxon tests showed no significant difference between Base 1 and Base 2 values (Z = 1, *p* = n.s.), and between the post-test and follow-up values (Z = −0.647, *p* = n.s.). However, there was a significant improvement in text memory from Base 2 to post-test (Z = 3.62, *p* < 0.001). The mean improvement from the averaged pre-treatment baselines (Base 1 and Base 2) to the averaged post-treatment measures (post-test and follow-up) in this period was an accuracy rate of 24.3% for correctly finding typing errors. In addition, a significant though numerically small increase in reading speed was found: there was a significant difference (according to the Friedman test) over the four time points (Figure 7a; X^2^ = 30.277, df = 3, *p* < 0.001). Subsequent paired comparisons using Wilcoxon tests showed no significant difference between Base 1 and Base 2 values (Z = −0.616, *p* = n.s.), and between the post-test and follow-up values (Z = −0.386, *p* = n.s.). However, there was a significant increase in the number of words read per minute from Base 2 to post-test (Z = −3.568, *p* < 0.001). The mean improvement in reading speed from the averaged pre-treatment baselines (Base 1 and Base 2) to the averaged post-treatment measures (post-test and follow-up) in this period was 12.9 WpM.

#### 3.2.3. Phone Numbers

**Figure 8:**
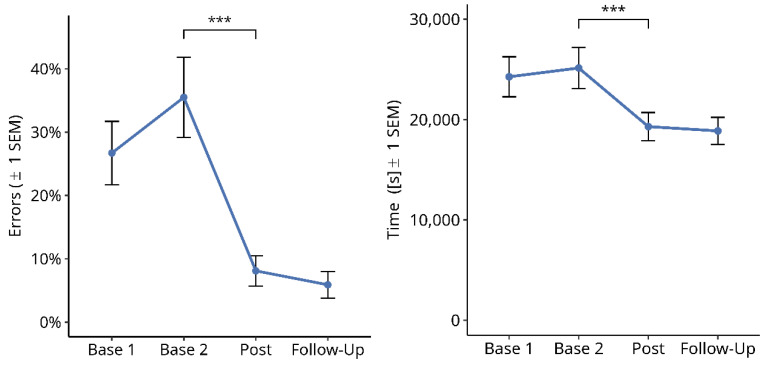
Effects of reading therapy on the number of errors in the phone number test (**a**) and performance time in the same task (**b**). The brackets and asterisks indicate significant statistical differences between conditions using two-tailed Wilcoxon test; (***) *p* ≤ 0.001.

Figure 8 shows the results for the percentage of errors in the phone number task (a) and the total performance time in seconds (b). A Friedman test over the four time points showed a significant difference for the percentage of errors in typed phone numbers (Figure 8a; X^2^ = 36.733, df = 3, *p* < 0.001). Subsequent paired comparisons using Wilcoxon tests showed no significant difference between Base 1 and Base 2 values (Z = 2.041, exact *p* = 0.041, n.s) or between the post-test and follow-up values (Z = −1.342. *p* = n.s.). However, there was a significant improvement from Base 2 to post-test (Z = −3.665, *p* < 0.001). The mean reduction in the percentage of errors from the averaged pre-treatment baselines (Base 1 and Base 2) to the averaged post-treatment measures (post-test and follow-up) was 24.1%. In addition, a significant difference in the total performance time was found over the four time points (Figure 8b; X^2^ = 34.398, df = 3, *p* < 0.001). Subsequent paired comparisons using Wilcoxon tests showed a small but significant prolongation of performance time between Base 1 and Base 2 (Z = −2.7, exact *p* = 0.006). No significant difference was found between the post-test and follow-up time points (Z = −2.058, exact *p* = 0.040, n.s). However, there was a significant reduction in performance time from Base 2 to post-test (Z = −3.724, *p* < 0.001). The mean reduction in performance time from the averaged pre-treatment baselines (Base 1 and Base 2) to the averaged post-treatment measures (post-test and follow-up) was 5617 ms (=22.7%).

#### 3.2.4. Subjective Symptom Load Questionnaire

**Figure 9:**
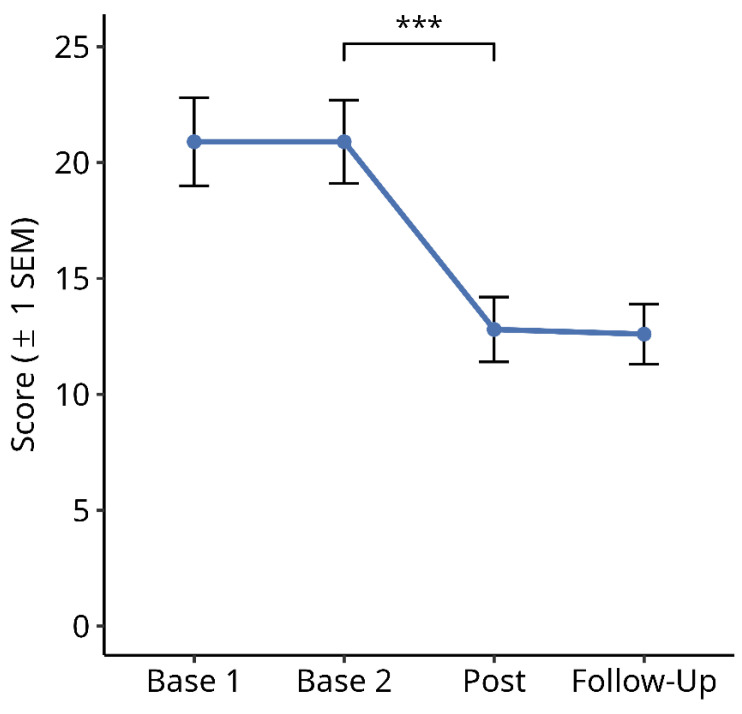
Effects of reading therapy on the Subjective Symptom Load Score. The brackets and asterisks indicate significant statistical differences between conditions using two-tailed Wilcoxon test; (***) *p* ≤ 0.001).

Figure 9 shows the results for the total score of the subjective symptom load questionnaire over the four time points. A Friedman test over the four time points showed a significant difference between the time points (Figure 9; X^2^ = 71.244, df = 3, *p* < 0.001). Subsequent paired comparisons using Wilcoxon tests showed no significant difference between Base 1 and Base 2 values (Z = −0.456, *p* = n.s.), and between the post-test and follow-up values (Z = −0.885, *p* = n.s.). However, there was a significant reduction in symptom load from Base 2 to post-test (Z = −4.553, *p* < 0.001). The mean reduction in the symptom load score from the averaged pre-treatment baselines (Base 1 and Base 2) to the averaged post-treatment measures (post-test and follow-up) in this period was 8.2 (=39%).

#### 3.2.5. Return to Work

We found that 63% (19 of 27) of the patients in our sample who were not already retired before their brain damage returned to their previous job. Approximately 50% of them worked part time (mean: 24.7 h per week) and another 50% worked full time (40 h per week) after the end of the reading therapy.

### 3.3. Further Analyses

We evaluated the possible influence of age, time since lesion, gender, visual acuity, visual field sparing and amount of treatment with the improvements (averaged pre-treatment scores vs. averaged post-treatment scores) using Spearman rank correlations. For this purpose, we computed a composite improvement score for all time-related measures (gain speed) in the reading tests (except the symptom load questionnaire) by adding the percentage of improvements in performance time. Likewise, we computed a composite score for all measures reflecting errors/correct solutions in the reading tasks (gain correct) by adding the percentage of improvements in all these measures.

Visual acuity for near or far distance was not significantly correlated with gain speed or gain correct (largest correlation: 0.339, *p* = n.s.). Likewise, time since lesion (in days) was not significantly correlated to either variable (largest correlation: −0.196, *p* = n.s.). Sex was not significantly correlated with the gain correct or gain time measures (largest point-biserial correlation: 0.281, *p* = n.s.). Age was significantly correlated with gain time (r = 0.529, *p* = 0.002), but not with gain correct (r = 0.180, *p* = n.s.). Finally, there were significant positive correlations between gain time and a reduction in symptom load after therapy (r = 0.423, *p* = 0.002), and between gain correct and the number of treatment sessions (r = 0.568, *p* = 0.002), i.e., treatment intensity.

## 4. Discussion

In summary, our sample of HD patients significantly improved in all measurements of our reading test battery, including the symptom load questionnaire, during treatment. In contrast, almost no improvements were seen in the pre-treatment interval before the reading therapy, which was twice as long as the therapy phase. Importantly, all improvements obtained during the therapy phase remained stable after the follow-up period, which lasted three times longer than the therapy phase. In some variables, small and statistically significant changes were seen either during the pre-treatment baseline or in the post-treatment follow-up period. However, most of these changes were numerically quite small in relation to the improvements obtained during treatment. Moreover, the time since the lesions was >4 months in 23 out of 27 patients (median: 219 days = 7 months), which highlights the effect of training instead of spontaneous recovery. Finally, the obtained improvements did not correlate with the time since lesion, nor with sex or visual acuity, but did correlate positively with age and treatment intensity. In the following sections, we will discuss our findings in more detail. We refrained from analyzing specific effects of drugs on reading progress as two recent Cochrane analyses found no evidence to support a therapeutic role of drugs on motor deficits [41] or visuospatial neglect [42] in individuals suffering from brain damage.

### 4.1. Basic Clinical Reading Tests and Relationship between Paper and Screen Reading Tasks

Similar to previous studies [10,20,43], we found significant improvements in reading speed in reading text on both paper and on a computer screen. The same holds true for the reduction in errors in both tasks. In fact, both tasks correlated with each other for reading speed and errors. This shows that our computerized reading therapy improved both reading on paper and reading on a computer screen in a similar way. This is important because it shows a positive transfer effect of our training on both types of reading tasks and not only on the reading tests shown on the PC screen. During daily life, reading text on paper and on various screens or optical displays is equally important, particularly at work. With respect to the size of the improvements, Virgili et al. [44] stated in their Cochrane review that the reading speed differences reported as improvements in several studies were 12 WpM. An increase in quality of life was reported by patients reaching or exceeding an improvement of 10 WpM. Here, we found gains of 20.8 WpM in paper-based reading and 9.8 WpM in reading from computer screens. In addition, the improvements in reading speed in the functional reading tasks ranged from 9.6 to 12.9 WpM or a 23% reduction in performance time in the phone number task. This highlights the efficiency of our treatment techniques with a rather limited treatment time (17.6 sessions = 1056 min on average). A future study could also differentiate if the effect of training depends on prior reading capability and digital familiarity as separate test variables. In particular, the affinity for the use of digital devices could be relevant for the adequate treatment of older patients.

### 4.2. Effects of Reading Therapy on Reading Tests and Possible Mechanisms

In previous studies [22,27,45], the training effects are accompanied by improvements in quality of life; here, we had a special focus on the question of whether reading training shows transfer effects for daily life activities. Similar to previous studies, we found that training effects led to an increase in self-reported quality of life measures [22,27,45]. Apart from these effects, we also found significant, though not large, improvements in reading speed and the number of errors made in single-word reading. This task was designed to mirror the RSVP therapy with single words. Although all patients also trained with the two other reading techniques, it appears reasonable to assume that the improvement in single-word reading was primarily related to this task. This technique proved to be particularly helpful for patients with slow reading and crowding problems in our sample (when several words are present, cf. [40]) as it reduces the complexity of the task.

It is interesting to note that nearly three-fourths of our HD patients were impaired in all functional reading tasks in the pre-treatment baseline tests, which underlines the tasks’ relevance in testing HD patients. The improvements in these functional reading tasks after therapy were impressive: we found a 79% improvement in text memory, and the patients even read faster in the parallel version of the post-test than in the pre-tests of this task. Hence, their improvement was not due to a slower reading strategy after treatment. Likewise, we found a 28% increase in the percentage of correctly identified typing errors. Again, their reading speed was faster than before in the parallel version of this test after treatment. Hence, the improvement in this task does not result from a slower reading strategy which then potentially allowed the patients to find more typing errors in the text. Finally, we found a 77% reduction in errors in the phone number task. Again, this did not result from slower reading in the parallel test of the text memory task after treatment.

To summarize, our HD patients learned to perform more accurately in all functional reading tasks and solved these tasks faster after therapy. This demonstrates that our chosen treatment combination led to a wide transfer of trained reading strategies (speed and accuracy) to untrained reading-related tasks and improved verbal memory. Since many patients with left posterior brain lesions [30] or head trauma [46] (both etiologies present in our sample) show verbal learning and verbal working memory deficits, reading therapy may be an interesting therapy option to improve these deficits, while improving reading performance at the same time.

### 4.3. Effects on Subjective Symptom Load, Treatment Intensity and Return to Work

We found a 39% reduction in subjective symptom load related to the reading disorder and associated neuro-visual deficits (i.e., blurred vision) after treatment, which correlated significantly with the speed increase (based on reduction in performance time) in all reading measures in our composite gain time score. Thus, speeding up reading reduces the subjective burden related to reading. Thus, reading therapy in HD might prove as an interesting option to reduce the considerable fatigue described in a recent Swedish study [6]. Hence, our treatment not only improved test scores but also reduced subjective impairments related to HD in daily life. Furthermore, the improvements in the global measure of correct performance (gain correct) correlated positively with the treatment time. Previous reading studies revealed no significant correlation between training intensity and reading speed [27]. However, our results showed that treatment intensity may be an important issue that should be addressed in forthcoming studies. A higher treatment intensity has been shown to result in a better outcome in motor therapies [47,48], and spatial neglect [49] and aphasia rehabilitation [50], but to the best of our knowledge, not for neuro-visual reading disorders. Finally, age positively correlated with the improvement in time (gain time). This probably reflects the fact that older subjects performed a bit slower in many timed reading tasks, so they could improve their reading time more during reading therapy. This shows that an older age is not a impairment for improving reading after a homonymous VFD.

Interestingly, we found that almost two-thirds of our sample (63%) managed to return to their previous job. Approximately 50% of them worked part time (mean: 24.7 h per week, range: 20–30), and another 50% worked full time (all worked 40 h per week) after the end of the reading therapy. This is, to our knowledge, the first study reporting return to work in HD patients. Return to work or its failure is an important aspect of participation that has been neglected up to now in many therapy studies with VFD patients [32] or those suffering from visual neglect [51].

## 5. Conclusions and Limitations

Despite the significant results reported here, several limitations should be mentioned. First, we used a single-subject baseline design without a control group, and the training sessions were not equally distributed for all patients. This approach was chosen because all patients were regular in-patients of our outpatient unit. Consequently, we refrained from allocating patients into a second (control) group which then eventually received no or another treatment or would have to wait a long time in a waiting group to receive treatment. This would have caused difficulties with the health insurance which paid for the treatment (but not for a “no” treatment). No other promising and effective reading therapy appeared suitable to us. Moreover, it should be pointed out that the improvements could also be related to compensatory processes that occur in the brain structures or to the drug treatment that the patients were receiving. As already discussed in the methods section, this seems to be unlikely due to the timing of the lesions and stable drug treatment. Following this argument, one would expect similar improvements between baseline time points or during follow-up testing. Uncertainties due to these factors are natural aspects in health service research [52], as clinical needs must be addressed along with the research. Despite these limitations, the improvements in the basic reading tasks are consistent with those previous studies on HD therapy [23], while the improvements in the functional reading tasks are novel and quite impressive. It also appears encouraging that nearly two-thirds of our patients returned to their prior (paid) work.

Second, our sample contained a mix of individuals with different types and sides of visual field defects in this sample who also suffered from brain damage with different etiologies. Again, while this may be a methodological weakness, because the sample is less homogenous, it is also a strength: it reflects a realistic (and not highly pre-selected) sample of patients with HD in a typical outpatient unit and, therefore, makes the generalization of findings to daily therapeutic practice in similar institutions easier. The etiologies in our sample are the most frequent and quite typical etiologies that cause HD [2,6]. To conclude, these novel findings may be integrated into a more effective reading therapy for HD patients that may promote reintegration into daily life and work.

Third, our patient sample was too small to perform a sub-analysis for distinct patient groups and treatment types. In a recent study, Kuester-Grueber et al. [27] showed that patients with right-sided VFD benefit from differential reading training compared to patients with left-sided VFD. In a future study, it should be investigated whether these specific subgroups benefit differently from our three treatment techniques. We hypothesize that patients with right-sided VFDs might benefit more from a floating text procedure, whereas patients with left-sided VFD, who have difficulties in finding the beginning of a text line, might benefit more from moving window text training. Fourth, we did not ask our patients for feedback regarding the treatment techniques and training intensity. In Kuester-Gruber et al. [27], the patients were asked to train twice a day for 30 min on 5 days per week. A part of their patients reported the training to be demanding or exhausting. To enhance training compliance, it would be interesting to record and consider the feedback from patients carefully in our future studies.

Fifth, our training should be tested to see whether it would also be effective as home-based training. For example, neuro-psychological care is not available in rural areas, and for patients with reduced mobility, it is difficult to visit an outpatient clinic several times a week for training. A home-based version could make the training available for a larger number of patients and lower the costs for health insurance [22,43,53]. However, this home-based training would have to been proven to lead to objective improvements in ADL (activity of daily living)-related tasks. For instance, there was no transfer effect in a study by Aimola et al. [22], and it was suggested that home-based training was less effective than the supervised equivalent.

## Figures and Tables

**Table 1:** Clinical and demographic data of 27 patients with hemianopic dyslexia (HD). L/R/BIL: left/right/bilateral. Mean values and ranges are given. For lesion location, multiple entries were used for different lesion locations. Md: median.

Variable	Score
Age (years)	50.2 (25–76), Md: 51
Sex (m/f)	16/11
Time since lesion (days)	638 (35–3258), Md: 219
Lesion side	
L/R/BIL-diffuse	10/10/7
Corrected visual acuity (%)	
Near distance (0.4 m)	0.97 (0.63–1.25)
Far distance (6 m)	0.93 (0.33–1.25)
Lesion location	
Frontal/temporal/parietal/occipital	0/5/8/19
Thalamus	2
Diffuse	7
Etiology	
Stroke	17
Tumor	4
Closed head trauma	6
Type of visual field defect	
Hemianopia (L, R, BIL)	4/4/4
Quadranopia (L, R)	3/3
Paracentral scotoma (L, R)	3/5
Diffuse small scotomas	1
Visual field sparing (mean °)	9.1 (1–35) Md: 4
Treatment hours (h)	17.6 (5–42) Md: 20

**Table 2:** Overview of the reading test battery [36] and Subjective Symptom Load Questionnaire. Pt. = patient. Pearson retest coefficients; *Spearman retest coefficients*; * Significance Level *p* < 0.01, two-tailed. cWpM/WpM: (correct) Words per Minute.

Subtest Description	Parallel Versions	Parameters	Retest Correlation Pearson Coefficient *Spearman Coefficient*
Text reading on paper, 180 words, Arial 12, black ink on white paper. Pt. is instructed to read the text on the table in front of him/her quickly and correctly aloud.	6	Correct words per minute read, errors	cWpM: 0.99 *, Errors: 0.98 * cWpM: 0.99 *, Errors: 0.94 *
2. Text reading PC-screen, 100 words, Arial 12, black on white. Pt. is instructed to read the text on the PC-screen quickly and correctly aloud.	3	Correct words per Minute read, errors	cWpM: 0.99 *, Errors: 0.98 * cWpM: 0.99 *, Errors: 0.86 *
3. Single Word reading, PC-screen 100 words, Arial 12, black on white. Pt. is instructed to read each single word on the PC-screen quickly and correctly aloud.	3	Correct words per Minute read, errors	cWpM:0.97 *, Errors: 0.98 * cWpM:0.95 *, Errors: 0.86 *
4. Text memory, 100 words, PC-screen, Arial 12, black on white, 5 numbers to remember from the text. Pt. is instructed to read the text on the PC-screen aloud and remember the numbers.	3	% correct remembered, Words per Minute read.	WpM: 0.99 *, correct: 0.92 * Wpm: 0.99 *, correct: 0.95 *
5. Find typing errors in text, PC-screen, 100 words, Arial 12, black on white, 7 errors to be detected. Pt. is instructed to find typing errors as quickly as possible in the text and indicate them.	3	% errors detected, Words per Minute	WpM: 0.87 *, correct: 0.96 * WpM: 0.86 *, correct: 0.93 *
6. Read and type phone numbers (8–13 digits long), PC-screen, Arial 12, black on white. Pt. is instructed to read 5 multi-digit numbers on the PC-screen and type them into the computer board as quickly as possible.	3	% correct, Total Time (seconds)	Time: 0.98 *, correct: 0.74 * Time: 0.96 *, correct: 0.82 *
7. Subjective Symptom Load Questionnaire (16 items, see Appendix A)	-	Summed score from 16 questions	Score: 0.99 * Score: 0.99 *

## Data Availability

The data in the study are available on request from the corresponding author. The data are not publicly available due to obligations of confidentiality of patient data.

## Appendix A

The Subjective Symptom Load Questionnaire—Reading and Reading-Related Tasks

0 = not at all 1 = somewhat 2 = partly 3 = mostly 4 = totally

I have had difficulties since the illness …. Score 0–4 for every question.

1. … when reading a newspaper

2. … when reading a book

3. … to hold the line when reading

4. … to find the beginning of the line when reading

5. … to look for the end of the line when reading

6. … when reading long words

7. … when reading on a computer/tablet

8. … when reading messages on a mobile phone/smartphone

9. … when reading signs in traffic

10. I leave out words/syllables when reading

11. I can no longer read for as long as I used to

12. … when writing

13. … when filling out checks/forms/letters/cards

14. … to recognize small writing or small numbers

15. My vision has been blurred since the illness

16. … recognize colors/colors look paler
